# Translation, Cultural Adaptation, Validation and Reliability of Persian Left Ventricular Dysfunction–36 Questionnaire

**DOI:** 10.34172/aim.2023.84

**Published:** 2023-10-01

**Authors:** Mehrbod Vakhshoori, Niloofar Bondariyan, Sayed Ali Emami, Niyousha Sadeghpour, Farbod Khanizadeh, Mahmood Emami, Davood Shafie

**Affiliations:** ^1^Heart Failure Research Center, Cardiovascular Research Institute, Isfahan University of Medical Sciences, Isfahan, Iran; ^2^Department of Clinical Pharmacy, School of Pharmacy, Shiraz University of Medical Sciences, Shiraz, Iran; ^3^Insurance Research Center, Tehran, Iran; ^4^Yazd Cardiovascular Research Institute, Yazd University of Medical Sciences, Yazd, Iran

**Keywords:** Heart failure, Iran, Left ventricular dysfunction, Quality of life, Reproducibility of results, Validation study

## Abstract

**Background:**

The left ventricular dysfunction 36 (LVD-36) questionnaire is considered to be a tool to assess the impact of left ventricle impairment on patients’ daily life. This methodological study was aimed to translate and assess the validity and reliability of the Persian draft of the LVD-36 questionnaire among Iranian heart failure (HF) patients.

**Methods:**

We recruited stable HF patients who referred to an outpatient heart clinic in Isfahan, Iran. The LVD-36 questionnaire was translated using the forward-backward method. Twenty HF patients were recruited for content validity assessment and were asked to express their opinions about the comprehensibility and meaningfulness of each item. We invited 14 experts to assess validity through content validity index (CVI) and content validity ratio (CVR). Reliability was assessed by Cronbach’s alpha and intraclass correlation coefficient (ICC), with the latter evaluated after invitation of the participants to complete the questionnaire for the second time.

**Results:**

The translation process was performed uneventfully without any significant alterations. A total of 150 HF patients were recruited to assess the reliability of the questionnaire in this study (age: 64.6±16 years, males: 58.6%). All items had acceptable CVI and CVR, ranging 0.85–1.00 and 0.57–1.00, respectively. Cronbach’s alpha was 0.971. All participants completed the questionnaire for the second time with no missing data. Test-retest reliability revealed an excellent ICC value of 0.981 (95% CI: 0.977–0.985).

**Conclusion:**

The Persian version of the LVD-36 questionnaire is a simple, valid and reliable tool for evaluating the impact of left ventricle impairment on the well-being of Iranian HF patients.

## Introduction

 Heart failure (HF) is defined as impaired ventricular filling or decreased heart ability to pump blood to meet systemic needs. It is divided into right and left HF with different signs and symptoms. Left ventricle dysfunction (LVD) is the most common cause of congestive HF in the context of several etiologies including hypertension, ischemic heart disease, diabetes, and structural problems of the heart.^1-3^ Left HF manifestations are paroxysmal nocturnal dyspnea, shortness of breath, orthopnea, and/or symptoms of volume overload (including increased abdominal girth, weight gain, leg swelling, or right upper quadrant pain due to liver congestion)^1^, which are disabling and negatively impact patients’ quality of life.^4^ HF is also known as a major growing medical and economic problem worldwide.^5,6^ According to the World Health Organization (WHO), the quality of life (QoL) is defined as: “*an individual’s perception of their position in life in the context of the culture and value systems in which they live and their goals, expectations, and standards, and concerns*”. This concept is influenced by various factors, including physical health, personal beliefs, psychological well-being, social relationships, and the individual’s interaction with salient features of their environment.^7^

 QoL is one of the most critical aspects of health which is affected by illnesses, and without a doubt, chronic diseases are more serious. They are increasingly becoming more and more common in recent decades.^4^ For patients with chronic conditions like mental disorders, asthma, kidney disease, chronic obstructive pulmonary disease, and congestive HF, evaluating the “quality of life” through health status measurement has become crucial in assessing the disease impact and effectiveness of treatment.^8^

 Questionnaires are used as tools to measure the QoL. Due to the variety of illnesses and their consequent morbidities, several QoL questionnaires are needed for further evaluation. In terms of congestive HF, several related questionnaires are available such as Chronic Heart Failure Assessment Tool (CHAT), Cardiac Health Profile congestive heart failure (CHPchf), Chronic Heart Failure Questionnaire (CHFQ), Kansas City Cardiomyopathy Questionnaire (KCCQ), Minnesota Living with Heart Failure Questionnaire (MLHFQ) and Quality of Life Questionnaire in Severe Heart Failure (QLQ-SHF), each of which has a specific application and is sometimes used in combination with or instead of others.^9-14^ However, using these questionnaires might be associated with difficulties regarding the required time in order to fill out the questionnaire completely as well as the total questionnaire length. Left Ventricular Disease Questionnaire (LVDQ) is a new short questionnaire designed for patients suffering from left ventricular impairment and can be completed in a timely manner. It is composed of 36 questions with true/false answers, and the results will be presented in the range 0–100%, which are the best and the worst scores, respectively.^15^ This questionnaire is also known as the left ventricular dysfunction questionnaire (LVD-36) and has been reported as a reliable and suitable tool for use to assess quality of life among patients with LVD.^16,17^

 The LVD-36 is originally in English and has been translated into other languages like Italian.^18^ Due to its importance and appliance and lack of a Persian version of the questionnaire, it needs to be translated into Persian for use in Iran. In this article, we decided to translate the questionnaire into Persian and examine its validity and reliability among Persian-speaking patients in Iran.

## Materials and Methods

###  LVD-36 Questionnaire

 The LVD-36 questionnaire is specifically designed to assess the impact of left ventricular impairment on the daily life of HF patients. It consists of 36 items, each with a true or false answering scale. The responses are summed, and the final score is reported as a percentage ranging from 0 (indicating the best possible score) to 100 (representing the worst possible score).^15^

###  Translation

 The translation process of the questionnaire, reported by Beaton et al, involved five phases.^19^ First, two bilingual translators, both native Persian speakers with fluency in English, were enlisted to independently translate the questionnaire from English to Persian. Only one of the translators had familiarity with medical terminology. They were asked to use meaningful and straightforward terms for translation at the second step. The first translated version was created after a final consensus between translators. Next, this Persian draft was translated backward to English. A meeting was held to discuss the pre-final version of the questionnaire with two translators and methodologists in the fourth step. Finally, we distributed the questionnaire among 20 patients who suffered from HF. They were asked to express their understanding of each question to the principal investigator. Their comments and probable questions were reviewed for possible modification of the questionnaire.

###  Validity

 A total of 14 experts, including 5 cardiologists, 2 general practitioners, 2 pharmacists, 1 statistician, and 4 nurses, were invited to assess the validity of the questionnaire. Each expert provided their feedback regarding the relevance, understandability, and comprehensibility of each item using a 4-point rating scale. The scale options were as follows: (1) not suitable, (2) suitable with minor adjustments, (3) suitable but requiring some modifications, and (4) very suitable. To calculate the CVI, the number of experts who rated an item as 3 or 4 was divided by the total number of experts. We considered the value of 0.80 as acceptable. Lower item CVI was defined as 0.70–0.79: re-evaluation required, < 70: candidate for omission.^20,21^ For CVR measurement, the experts were required to declare their opinions about each provided item on the questionnaire with a 3-item scale (essential, important but not essential, not essential). A ratio of at least 0.57 was defined as acceptable.^22^

###  Reliability

 We evaluated the internal consistency of the questionnaire using Cronbach’s alpha coefficient, which is a measure of reliability. The interpretation of Cronbach’s alpha values is as follows: ≥ 0.9 indicates excellent internal consistency, 0.8–0.9 indicates good internal consistency, 0.7–0.79 indicates acceptable internal consistency, 0.6–0.69 indicates questionable internal consistency, 0.5–0.59 indicates poor internal consistency, and < 0.5 indicates unacceptable internal consistency.^23^ We measured test-retest reliability by asking the recruited participants to answer the questionnaire questions for the second time after approximately two weeks, and the calculated ICC was characterized as follows: ≥ 0.75: excellent, 0.4–0.75: fair to good, and < 0.4: poor.^24^

###  Floor and Ceiling Effect

 We evaluated the presence of floor and ceiling effects by examining whether more than 15% of the study population obtained the lowest and highest scores, respectively.

###  Statistical Analysis

 The distribution of the participants’ answers on each questionnaire item was reported as frequency (percentage). Also, mean ± standard deviation (SD) was used to report total scores. All analyses were done with the Statistical Package for Social Sciences (SPSS) version 22 (IBM Corp., Armonk, NY, USA).

###  Ethical Considerations

 The Ethics Committee of Isfahan University of medical sciences (IUMS) approved this study (IR.MUI.MED.REC.1400.562). At the start of the study, the principal investigator provided a comprehensive explanation of the main objectives to each participant. Ample time was given to participants to ask any questions they had about the project. Moreover, all individuals were assured that their personal information would remain confidential and not divulged publicly. Finally, each patient was provided with a written consent form to sign.

## Results

###  Questionnaire Performance

**Figure 1:**
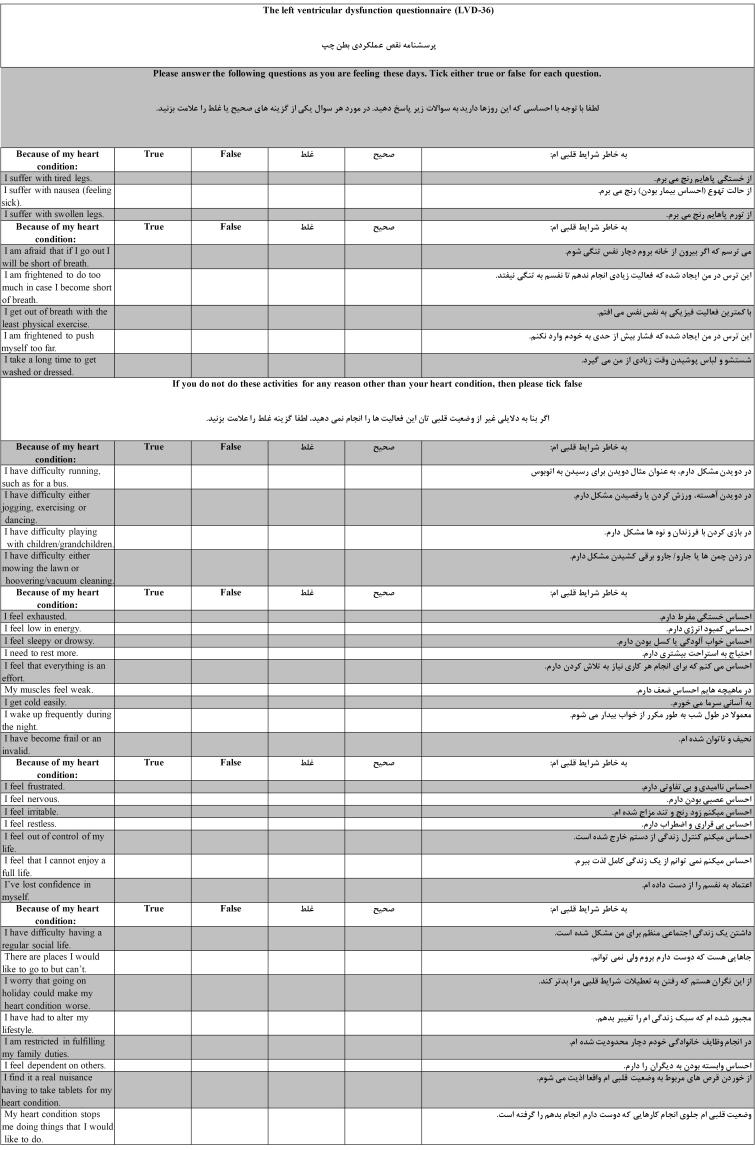
Persian and Original Version of LVD-36 Questionnaire

 Out of 20 HF patients who fulfilled the questionnaire, most declared that all the questions were easily understandable in the translated version and no major changes were made in the final questionnaire. Figure 1 shows the Persian version of the LVD-36 questionnaire in comparison to the original one. The final Persian draft of the LVD-36 questionnaire is presented in Supplementary file 1.

###  Questionnaire Validity

Table 1 presents data on the CVI and CVR of each questionnaire item. All questions had CVI ranging from 0.85 to 1.00. Moreover, each item showed acceptable CVR (minimum: 0.57, maximum: 1.00).

**Table 1:** Validity Indices of the Persian Version of the Left Ventricle Dysfunction-36 (LVD-36) Questionnaire

**Questions**	**Content Validity Index**	**Content Validity Ratio**
Item 1	0.85	0.86
Item 2	0.92	0.57
Item 3	1.00	0.86
Item 4	0.92	0.71
Item 5	1.00	0.57
Item 6	1.00	0.71
Item 7	1.00	0.71
Item 8	0.85	0.86
Item 9	0.85	0.57
Item 10	0.85	1.00
Item 11	0.92	0.86
Item 12	0.92	1.00
Item 13	1.00	0.86
Item 14	0.92	0.86
Item 15	0.85	0.86
Item 16	0.85	0.57
Item 17	1.00	0.86
Item 18	1.00	0.71
Item 19	0.92	1.00
Item 20	0.85	0.71
Item 21	1.00	1.00
Item 22	0.92	1.00
Item 23	0.92	0.71
Item 24	1.00	1.00
Item 25	0.92	1.00
Item 26	1.00	0.57
Item 27	0.85	1.00
Item 28	1.00	1.00
Item 29	1.00	0.71
Item 30	0.85	0.86
Item 31	0.92	1.00
Item 32	1.00	0.71
Item 33	0.85	1.00
Item 34	0.92	1.00
Item 35	0.85	1.00
Item 36	0.92	0.57

###  Participants’ Characteristics

 A total of 150 HF patients completed the questionnaire on two separate occasions, with a two-week interval between each administration (age: 64.6 ± 16 years, males: 58.6%). The distribution of their answers based on each item on the first and second round of questionnaire completion is shown in Table 2. The mean LVD score after the first completion was 94.09 ± 16.3%.

**Table 2:** Distribution of Respondents’ Answers during the First and Second Round of Completing the Persian Left Ventricle Dysfunction-36 (LVD-36) Questionnaire (n = 150)

**Questions**	**First Round**		**Second Round**	
	**Yes (%)**	**No (%)**	**Yes (%)**	**No (%)**
Item 1	140 (93)	10 (15)	117 (78)	33 (22)
Item 2	141 (93)	9 (15)	138 (92)	12 (15)
Item 3	107 (71)	43 (29)	67 (45)	83 (55)
Item 4	136 (91)	14 (9)	139 (93)	11 (15)
Item 5	140 (93)	10 (15)	140 (93)	10 (15)
Item 6	142 (95)	8 (5)	142 (95)	8 (5)
Item 7	142 (95)	8 (5)	142 (95)	8 (5)
Item 8	133 (89)	17 (11)	134 (90)	16 (10)
Item 9	147 (98)	3 (2)	147 (98)	3 (2)
Item 10	136 (91)	14 (9)	138 (92)	12 (15)
Item 11	135 (90)	15 (10)	134 (90)	16 (10)
Item 12	140 (93)	10 (15)	141 (93)	9 (15)
Item 13	144 (96)	6 (4)	141 (93)	9 (15)
Item 14	146 (97)	4 (3)	146 (97)	4 (3)
Item 15	145 (97)	5 (3)	145 (97)	5 (3)
Item 16	145 (97)	5 (3)	143 (95)	7 (5)
Item 17	145 (97)	5 (3)	145 (97)	5 (3)
Item 18	147 (98)	3 (2)	146 (97)	4 (3)
Item 19	147 (98)	3 (2)	144 (96)	6 (4)
Item 20	145 (97)	5 (3)	142 (95)	8 (5)
Item 21	144 (96)	6 (4)	141 (93)	9 (15)
Item 22	145 (97)	5 (3)	144 (96)	6 (4)
Item 23	138 (92)	12 (15)	138 (92)	12 (15)
Item 24	141 (93)	9 (15)	141 (93)	9 (15)
Item 25	143 (95)	7 (5)	143 (95)	7 (5)
Item 26	141 (93)	9 (15)	141 (93)	9 (15)
Item 27	141 (93)	9 (15)	141 (93)	9 (15)
Item 28	141 (93)	9 (15)	141 (93)	9 (15)
Item 29	139 (93)	11 (15)	139 (93)	11 (15)
Item 30	142 (95)	8 (5)	142 (95)	8 (5)
Item 31	142 (95)	8 (5)	142 (95)	8 (5)
Item 32	144 (96)	6 (4)	144 (96)	6 (4)
Item 33	143 (95)	7 (5)	143 (95)	7 (5)
Item 34	144 (96)	6 (4)	144 (96)	6 (4)
Item 35	145 (97)	5 (3)	145 (97)	5 (3)
Item 36	145 (97)	5 (3)	145 (97)	5 (3)
Total score	94.09 ± 16.3%		92.7 ± 16.3%	

###  Questionnaire Reliability

 The Cronbach’s alpha coefficient of the current Persian version of the LVD-36 questionnaire was 0.971, showing excellent internal consistency. The ICC of the questionnaire two weeks after the second round of completion was found to be 0.981 (95% confidence interval [CI]: 0.977-0.985). The reliability indices of questionnaire items are shown in Table 3. The minimum and maximum values of corrected item-total correlation (CITC) were 0.234 (item 18) and 0.943 (items 30 and 31), respectively. Also, all items contributed desirably to internal reliability in a way that deletion of questions did not significantly increase the value of Cronbach’s alpha.

**Table 3:** Reliability Indices of the Persian Version of the Left Ventricle Dysfunction-36 (LVD-36) Questionnaire

**Questions**	**Corrected Item-Total Correlation**	**Cronbach's Alpha If Item Deleted**
Item 1	0.621	0.970
Item 2	0.789	0.969
Item 3	0.387	0.974
Item 4	0.674	0.970
Item 5	0.727	0.970
Item 6	0.753	0.970
Item 7	0.775	0.970
Item 8	0.748	0.970
Item 9	0.625	0.970
Item 10	0.662	0.970
Item 11	0.614	0.971
Item 12	0.640	0.970
Item 13	0.535	0.971
Item 14	0.696	0.970
Item 15	0.690	0.970
Item 16	0.690	0.970
Item 17	0.637	0.970
Item 18	0.234	0.971
Item 19	0.483	0.971
Item 20	0.677	0.970
Item 21	0.748	0.970
Item 22	0.499	0.971
Item 23	0.831	0.969
Item 24	0.672	0.970
Item 25	0.740	0.970
Item 26	0.902	0.969
Item 27	0.902	0.969
Item 28	0.713	0.970
Item 29	0.884	0.969
Item 30	0.943	0.969
Item 31	0.943	0.969
Item 32	0.784	0.970
Item 33	0.883	0.969
Item 34	0.852	0.969
Item 35	0.750	0.970
Item 36	0.717	0.970

###  Floor and Ceiling Effect

 No instances of the highest or lowest scores were observed in our study population, indicating the absence of both floor and ceiling effects.

## Discussion

 In this study, we aimed to investigate the validity and reliability of the Persian version of the LVD-36 questionnaire. We found that this questionnaire could appropriately assess the main health issues of Iranian patients suffering from HF. The prevalence of HF has increased during recent years, and assessing its effect on patients’ daily life could significantly aid in better implementation of therapeutic strategies.

 This questionnaire was first developed based on 179 items collected from multiple sources, including prior questionnaires, published records, patient interviews, and physician consultations. After the completion of all items by 139 individuals suffering from left ventricular impairment and further analysis, 36 items were finally selected, and the final version of the LVD-36 questionnaire was officially introduced.^25,26^

 We used one of the most common translation methods, namely the forward-backward method. The dual-panel is another method using two different phases, including a bilingual panel and a lay panel. The first panel conducts the initial translation of the questionnaire, while the second group, consisting of monolingual individuals, is asked to provide feedback on the clarity and comprehensibility of the translated version.^27,28^ It has been reported that the dual-panel method has fewer missing items during the translation process. However, this method is more time-consuming and might be associated with difficulties to perform thoroughly.^29^ Moreover, it has been recommended that the translated draft of the questionnaire is needed to be combined with backward translation.^27,30^ Therefore, we decided to use the common forward-backward translation method. Also, our findings revealed that there was not any missing item during translation of the original LVD-36 questionnaire. The respondents declared that the questionnaire had appropriately understandable questions and needed approximately five minutes for completion.

 Our results in terms of reliability, as an index to evaluate the ability of the questionnaire for accurate measurement, revealed a Cronbach’s alpha of 0.971. Also, after completing the questionnaire for the second time by all participants, the ICC value was found to be 0.981 (95% CI: 0.977–0.985), that indicates this questionnaire is a reliable tool for assessing general health among HF patients. O’Leary and Jones recruited 60 patients with chronic left ventricle dysfunction to assess the reliability of the LVD-36 questionnaire. All patients completed the questionnaire for the first time, with 53 subjects re-completing one to three weeks later. The mean time reported to be taken in order to complete the questionnaire was approximately 5 minutes. The internal consistency of the LVD-36 questionnaire was 0.95 with a similar ICC (0.95). They suggested that this questionnaire has a high degree of reliability with unambiguous wording.^15^ Moreover, Miani et al recruited 50 patients with congestive HF and translated the original questionnaire to the Italian language. After assessing reliability, they suggested that this instrument was a reliable tool among Italian patients.^18^ The floor and ceiling effect values in another study were 0 and 3%, respectively.^15^ However, we did not observe this effect among our responders.

 This study demonstrates that the Persian version of the LVD-36 questionnaire showed good content validity, indicating that the instrument accurately measured its intended constructs. Additionally, a comparison between the LVD-36 and the short-form 36 (SF-36) questionnaire revealed stronger correlations in physical well-being and weaker correlations in psychosocial well-being.^15^ Another study revealed that the Italian version of the LVD-36 questionnaire correlated well with the MLHF questionnaire.^18^

 To the best of our knowledge, this study is the first in the literature to assess the reliability and validity of the Persian-translated LVD-36 questionnaire among Iranian HF patients. Completing by a large number of study samples twice without any missing data could be considered another strength of the current study. However, some limitations also exist. This study was implemented in a single center, and our findings might not be generalizable to other Iranian individuals living in other Iranian cities. We also did not assess the educational levels of the enrolled participants, which might have affected our outcomes.

## Conclusion

 In conclusion, the Persian version of the LVD-36 questionnaire is as accurate and reliable as the original version. It is simple and understandable, has a high reliability and validity index, and can be used in clinical settings to assess the quality of life and general health status of Iranian HF patients.

## Supplementary Files

Supplementary file 1 contains Figure S1.
