# Supplementary material for: Translation, Cultural Adaptation, Validation and Reliability of Persian Left Ventricular Dysfunction–36 Questionnaire
Source: Arch Iran Med. 2023 Oct 1;26(10):575–81. doi: 10.34172/aim.2023.84 (PMC10862096; doi:10.34172/aim.2023.84)
Supplement: Supplementary file 1 — contains Figure S1. [file aim-26-575-s001.pdf]

Arch Iran Med. October 2023;26(10):575-581

10.34172/aim.2023.84

Supplementary file 1

پرسشنامه نقص عملکردی بطن چپ

لطفا با توجه با احساسی که این روزها دارید به سوالات زیر پاسخ دهید. در مورد هر سوال یکی از گزینه های صحیح یا غلط را علامت بزنید.

به خاطر شرایط قلبی ام:	صحیح	غلط
از خستگی پاهایم رنج می برم.		
از حالت تهوع (احساس بیمار بودن) رنج می برم.		
از تورم پاهایم رنج می برم.		
به خاطر شرایط قلبی ام:	صحیح	غلط
می ترسم که اگر بیرون از خانه بروم دچار نفس تنگی شوم.		
این ترس در من ایجاد شده که فعالیت زیادی انجام ندهم تا نفسم به تنگی نیفتد.		
با کمترین فعالیت فیزیکی به نفس نفس می افتم.		
این ترس در من ایجاد شده که فشار بیش از حدی به خودم وارد نکنم.		
شستشو و لباس پوشیدن وقت زیادی از من می گیرد.		
به خاطر شرایط قلبی ام:	صحیح	غلط
در دویدن مشکل دارم، به عنوان مثال دویدن برای رسیدن به اتوبوس		
در دویدن آهسته، ورزش کردن یا رقصیدن مشکل دارم.		
در بازی کردن با فرزندان و نوه ها مشکل دارم.		
در زدن چمن ها یا جارو/ جارو برقی کشیدن مشکل دارم.		
به خاطر شرایط قلبی ام:	صحیح	غلط
احساس خستگی مفرط دارم.		
احساس کمبود انرژی دارم.		
احساس خواب آلودگی یا کسل بودن دارم.		
احتیاج به استراحت بیشتری دارم.		
احساس می کنم که برای انجام هر کاری نیاز به تلاش کردن دارم.		
در ماهیچه هایم احساس ضعف دارم.		
به آسانی سرما می خورم.		
معمولا در طول شب به طور مکرر از خواب بیدار می شوم.		
نحیف و ناتوان شده ام.		
به خاطر شرایط قلبی ام:	صحیح	غلط
احساس ناامیدی و بی تفاوتی دارم.		
احساس عصبی بودن دارم.		
احساس میکنم زود رنج و تند مزاج شده ام.		
احساس بی قراری و اضطراب دارم.		
احساس میکنم کنترل زندگی از دستم خارج شده است.		
احساس میکنم نمی توانم از یک زندگی کامل لذت ببرم.		
اعتماد به نفسم را از دست داده ام.		
به خاطر شرایط قلبی ام:	صحیح	غلط
داشتن یک زندگی اجتماعی منظم برای من مشکل شده است.		
جاهایی هست که دوست دارم بروم ولی نمی توانم.		
از این نگران هستم که رفتن به تعطیلات شرایط قلبی مرا بدتر کند.		
مجبور شده ام که سبک زندگی ام را تغییر بدهم.		
در انجام وظایف خانوادگی خودم دچار محدودیت شده ام.		
احساس وابسته بودن به دیگران را دارم.		
از خوردن قرص های مربوط به وضعیت قلبی ام واقعا اذیت می شوم.		
وضعیت قلبی ام جلوی انجام کارهایی که دوست دارم انجام بدهم را گرفته است.		

Figure S1. Persian Version of the LVD-36 Questionnaire.
